# Systematic Analysis and Comparison of Nucleotide-Binding Site Disease Resistance Genes in a Diploid Cotton *Gossypium raimondii*

**DOI:** 10.1371/journal.pone.0068435

**Published:** 2013-08-06

**Authors:** Hengling Wei, Wei Li, Xiwei Sun, Shuijin Zhu, Jun Zhu

**Affiliations:** 1 Key Laboratory of Crop Germplasm, Department of Agronomy, Zhejiang University, Hangzhou, Zhejiang, China; 2 State Key Laboratory of Cotton Biology, Cotton Research Institute, Chinese Academy of Agricultural Sciences, Anyang, Henan, China; Pennsylvania State University, United States of America

## Abstract

Plant disease resistance genes are a key component of defending plants from a range of pathogens. The majority of these resistance genes belong to the super-family that harbors a Nucleotide-binding site (NBS). A number of studies have focused on NBS-encoding genes in disease resistant breeding programs for diverse plants. However, little information has been reported with an emphasis on systematic analysis and comparison of NBS-encoding genes in cotton. To fill this gap of knowledge, in this study, we identified and investigated the NBS-encoding resistance genes in cotton using the whole genome sequence information of *Gossypium raimondii*. Totally, 355 NBS-encoding resistance genes were identified. Analyses of the conserved motifs and structural diversity showed that the most two distinct features for these genes are the high proportion of non-regular NBS genes and the high diversity of N-termini domains. Analyses of the physical locations and duplications of NBS-encoding genes showed that gene duplication of disease resistance genes could play an important role in cotton by leading to an increase in the functional diversity of the cotton NBS-encoding genes. Analyses of phylogenetic comparisons indicated that, in cotton, the NBS-encoding genes with TIR domain not only have their own evolution pattern different from those of genes without TIR domain, but also have their own species-specific pattern that differs from those of TIR genes in other plants. Analyses of the correlation between disease resistance QTL and NBS-encoding resistance genes showed that there could be more than half of the disease resistance QTL associated to the NBS-encoding genes in cotton, which agrees with previous studies establishing that more than half of plant resistance genes are NBS-encoding genes.

## Introduction

Plant diseases can dramatically reduce crop yield and quality. Pathogens and pests are the two major factors causing plant diseases. Disease resistance genes (R-genes) play a critical role in plant disease-resistance detection and they response to several pathogens and pests, including viruses, bacteria, fungi, nematodes, and insects. After isolating the first plant resistance gene, Hm1, from maize (*Zea mays*) in 1992 [1], more than 70 R genes have been cloned; most of the cloned genes are nucleotide binding sites (NBS) genes [2] containing NBS domain and forming one of the largest plant resistance gene families. To understand the structure, evolution, and molecular basis of the NBS genes, Genome-wide identification and analysis of NBS genes have been performed on many plants [3]–[17]. In order to increase crop yield and quality, it is important to conduct genomic studies of NBS genes to better understand the mechanism of plant susceptibility and resistance.

The NBS is a signaling domain responsible for ATP/GTP binding and hydrolysis in plants defense system [18]. Based on the structures of the N-terminal domain, the NBS protein family has been divided into two distinct subgroups [19]. One subgroup, which has been shown to be involved in determining the specificity of resistance and signaling [20], [21], has an N-terminal region homologous to the Toll/interleukin-1 receptor (TIR); whereas the other subgroup, which has been inferred, might be relative to protein-protein interactions and signaling [22], [23], has a coiled-coil (CC) domain. The NBS domains of TIR and CC proteins contain five strictly ordered motifs including P-loop, kinase-2, kinase-3a, GLPL, and MHDL. An obvious separation between TIR proteins and CC proteins is observed in most plant phylogenetic trees of the disease resistance NBS domains [11]–[14], [16], which suggests that the TIR and CC subgroups may have different evolutionary histories.

NBS genes were usually found as clusters of tandem repeats that mostly derived from tandem duplication events and caused a higher proportion of duplicated genes in NBS gene family [3], [10], [17], [24]–[26]. Although all NBS genes tend to retain the conserved domains, considerable variations of these genes were detected in different species or same species. Duplication analysis of NBS genes in soybean implied that the rapid evolution of NBS genes is due to the combined effects of diversifying selection and frequent sequence exchanges [6]. In contrast to genes expansion caused by duplication, genes contraction caused by high frequency of NBS gene loss after whole-genome duplication was also detected in plants [27]. According to the research on *Arabidopsis* and gramineous plants [26], [28], Chen and Li proposed that expansion or contraction of NBS genes might be a basic strategy adopted by plants to protect themselves from the changing species-specific pathogen spectrum. However, little is known about the distribution, expansion, and contraction of NBS resistance genes in cotton genome; a genome wide analysis of NBS resistance gene on cotton will increase the knowledge and understanding of the evolution of NBS resistance genes.

Cottons, which belong to the genus of *Gossypium*, are the most economically important crop plants worldwide. Additionally, cottons are also used as a model species for evolutionary studies of polyploidy plants. The genus of *Gossypium* contains more than 50 species (including 5 tetraploid and over 45 diploid species) that probably originated from a common ancestor approximately 5–10 million years ago [29]. The tetraploid cotton species, such as *G. hirsutum* and *G. barbadense*, are the production of the allopolyploidization event of A and D genome that occurred approximately 1–2 million years ago [30]. In order to provide a source of candidate genes important for the genetic improvement of cotton quality and productivity, a diploid draft sequence of the putative D-genome donor, *G. raimondii*, has been created [31]. With access to this full genome sequence, we can annotate the NBS resistance genes in a genome wide level, and offer more information about the disease resistance genes.

The main purpose of the present study was to use the available diploid cotton genome sequences to identify candidates for NBS disease resistance genes of cotton. At the same time, we also detected these genes from various aspects, including the characterization of functional domain and motifs, the distribution across the genome, the duplication events among them, and the phylogenetic relationship between NBS resistance genes belonging to cotton and other plants. These analyses provided some genome-level insights about resistance genes of cotton, and can help to accelerate the disease-resistant breeding of cotton.

## Materials and Methods

### Identification and classification of NBS-encoding resistance genes

The diploid cotton (*Gossypium raimondii*) gene information was provided by The Cotton Research Institute, Chinese Academy of Agricultural Sciences (http://cgp.genomics.org.cn/). This information contains annotations of 40,976 protein-coding genes in the *G. raimondii* genome [31]. To identify NBS-encoding resistance genes in the diploid cotton, first, a set of 113 manually curated reference disease resistance genes was selected from plant resistance gene database (http://www.prgdb.org) [32] (Table S3), and then the protein sequence of the 40,976 protein-coding genes were subsequently checked for sequence homology with at least one resistance protein contained in the reference dataset using the BLAST algorithm with a stringent e-value cut-off of 1×10^−15^; in the second step, domain analysis of all the BLAST hits were performed using InterProScan version 5RC2 [33] with standard options and the InterPro database release 7.2; in the third step, the genes with NBS domain were filtered out according to the NBS domain annotation (PF00931) given by the Pfam (Protein family) database [34].

The N-termini and LRR regions were initially targeted to classify the NBS genes using the Pfam database, SMART protein motif analyses, and the MARCOIL program. The MARCOIL [35] program was used to detect the CC domain, while Pfam database [34] and SMART protein motif analysis (Simple Modular Architecture Research Tool) [36] were used to detect the TIR and LRR domains.

### Analysis of gene duplication

Gene duplication was identified when the following conditions are fulfilled: (1) the alignment covered more than 70% of the longer gene, (2) the region of identity between them is larger than 70% of the alienable region, and (3) only one duplication event was counted for tightly-linked genes [37]. Gene families were defined as groups in which each gene displays identity to at least one other member of the group.

### Sequence alignment and phylogenetic analysis

Multiple alignments of amino acid sequences were performed using ClustalW [38] with default options; a subsequent manual alignment correction was accomplished by using MEGA 5.05 [39]. Phylogenetic trees were constructed by means of the bootstrap neighbor-joining (NJ) method and a Kimura 2-parameter model that are provided by MEGA 5.05. The stability of internal nodes was assessed by bootstrap analysis with 1,000 replicates. These trees were subsequently used to analyze the evolutionary relationships among NBS disease-resistant genes.

### Disease resistance QTL adjacent to NBS-encoding genes

The cotton disease resistant QTL for *Verticillium* wilt, *Fusarium* wilt, and Root-Knot Nematode were retrieved from previous studies [40]–[45]. The tetraploid genome physical locations of these QTL were also retrieved from the same studies [40]–[45] and Cotton Marker Database (http://www.cottonmarker.org). The primers of the markers corresponding to these QTL were retrieved from Cotton Marker Database too. The diploid genome physical locations of these QTL were analyzed by electronic PCR [46] using the primers of their corresponding markers with a size cut-off of 80–700 bp. The distance between the marker and the NBS-encoding genes were accounted by their positions on the diploid cotton genome.

## Results

### Identification and classification of NBS-encoding genes

Using the most recent diploid cotton (*G. raimondii*) genome sequence data provided by Cotton Research Institute, Chinese Academy of Agricultural Sciences, a total of 355 NBS disease resistance genes were identified within the assembled sequence of *G. raimondii*. Analysis of the 355 genes with an NBS structure (based on the Pfam database) identified 163 genes with highly conserved NBS regions and a complete open reading frame (ORF). These 163 genes were classified as regular NBS genes. The remaining 192 NBS-encoding genes are notably different from the regular NBS-encoding genes as a result of having only some of the conserved NBS motifs, and were therefore classified as non-regular NBS genes.

In order to know the structural divergence and conserved motifs shared among the NBS genes, amino acid alignment of NBS domains for the NBS proteins was analyzed. In the regular NBS gene subgroups, all the five conserved motifs regions (P-loop, Kinase2, Kinase3, GLPL and MHDL) can be clearly revealed in the NBS domain by amino acid alignment (Figure S2) and MEME analysis. In the non-regular NBS gene subgroups, only some of the five motifs can be revealed in the NBS domain, but these motifs follow the same order shown on the regular NBS domain; for example, if only three motifs are revealed, they should be: 1) P-loop, Kinase2 and Kinase3, 2) Kinase2, Kinase3 and GLPL, or 3) Kinase3, GLPL and MHDL (Figure S3).

In order to classify these NBS genes with more detail, we also checked other kind of their domains besides NBS domain. In the next few lines of this paragraph, we will give a detailed description of the methods about how to classify these NBS genes and how to abbreviate the subgroups in which genes were classified. If CC domain was detected at N-termini under a threshold of 0.90, the CC domain would be coded as “C”; otherwise, the CC domain would be coded as “c” if CC domain was detected at N-termini under a threshold of 0.50, 0.10, or 0.02. If TIR domain was detected at N-termini, the TIR domain would be coded as “T”. If no domain was detected at N-termini and the sequence before NBS domain still contains more than 100 amino-acid residues, the N-termini would be coded as “X”; otherwise, no code would be given to these N-termini. If LRR domain was detected after NBS domain, the LRR domain would be coded as “L”. If no domain was detected after NBS domains and the sequence after NBS domain still contains more than 100 amino-acid residues, this part would be coded as “X”; otherwise, no code would be given to this part. The individual codes mentioned above can be combined as letter codes or abbreviations for the subgroups of the NBS genes shown in Table 1, such letter codes can indicate the structures of the genes too. For example, a CNL code means that genes in this subgroup contain: a CC domain at N-termini under a threshold of 0.90 (C), a NBS domain (N), and a LRR domain after NBS domain (L).

**Table 1:** Subgroup numbers of NBS-encoding genes in *G. raimondii*.

Predicted protein domains	Letter code	Number of genes	Percentage of total genes (%)	
Regular				
CC domain N-terminal	CN	3	0.84	13.52
	CNL	24	6.76	
	CNX	21	5.91	
	cNL	57	16.06	21.97
	cNX	21	5.92	
TIR domain N-terminal	TN	3	0.85	7.61
	TNL	19	5.35	
	TNX	5	1.41	
other kind of N-terminal	N	1	0.28	2.82
	NL	7	1.97	
	NX	2	0.56	
Total regular NBS genes	163		45.92	
Non-regular				
CC domain N-terminal	CN	15	4.23	12.11
	CNL	11	3.10	
	CNX	17	4.79	
	cN	21	5.92	14.37
	cNL	11	3.10	
	cNX	19	5.35	
TIR domain N-terminal	TN	6	1.69	10.70
	TNL	22	6.20	
	TNX	10	2.82	
Other kind of N-terminal	XN	2	0.56	2.25
	XNL	4	1.13	
	XNX	2	0.56	
	N	9	2.54	14.65
	NL	21	5.92	
	NX	22	6.20	
Total non-regular NBS genes	192		54.08	
Total NBS genes	355			

Firstly, based on sequence differences in the N-termini, the regular NBS disease resistance genes were classified into three groups (Table 1): CC domain N-terminal, TIR domain N-terminal, and other kind of N-terminal. Secondly, according to the different thresholds of MARCOIL and the sequence after NBS domain, CC domain N-terminal group was classified into five subgroups CN, CNL, CNX, cNL, and cNX. Similarly, based on the sequence after NBS domain, TIR domain N-terminal group was classified into three subgroups: TN, TNL, and TNX. Also in the same way, according to the sequence after NBS domain, other kind of N-terminal group was classified into three subgroups: N, NL, and NX.

Following the same standard procedures used to classify the regular NBS disease-resistance genes into groups and subgroups, the non-regular NBS disease-resistance genes were classified (Table 1). Three groups were generated: CC domain N-terminal, TIR domain N-terminal, and other kind of N-terminal. In the case of the subgroups, the group with CC domain was classified into six subgroups: CN, CNL, CNX, cN, cNL, and cNX; the group with TIR domain was classified into three subgroups: TN, TNL, and TNX; finally, the group with other kind of N-terminal was classified into six subgroups: XN, XNL, XNX, N, NL, and NX.

With respect to the regular NBS genes, under the lowest (0.02) threshold, there were 126 genes possessing CC domains, but only 48 out of these 126 genes (38.10%) could be detected possessing CC domains under a threshold of 0.9. Twenty seven genes were detected with TIR motif (3 TN, 19 TNL, and 5 TNX). The remaining 10 regular NBS genes were classified as other kind of N-terminal group (1N, 7NL, and 2 NX).

In the case of the non-regular NBS genes, under the lowest threshold, there were 93 genes possessing CC domains, but only 42 genes (45.16%) could be detected having CC domains under a threshold of 0.9. Thirty eight genes were detected with TIR motif (6 TN, 22 TNL, and 10 TNX). The remaining 61 non-regular NBS genes were classified as other kind of N-terminal group (2XN, 4XNL, 2XNX, 10N, 21NL, and 22 NX).

**Figure 1:**
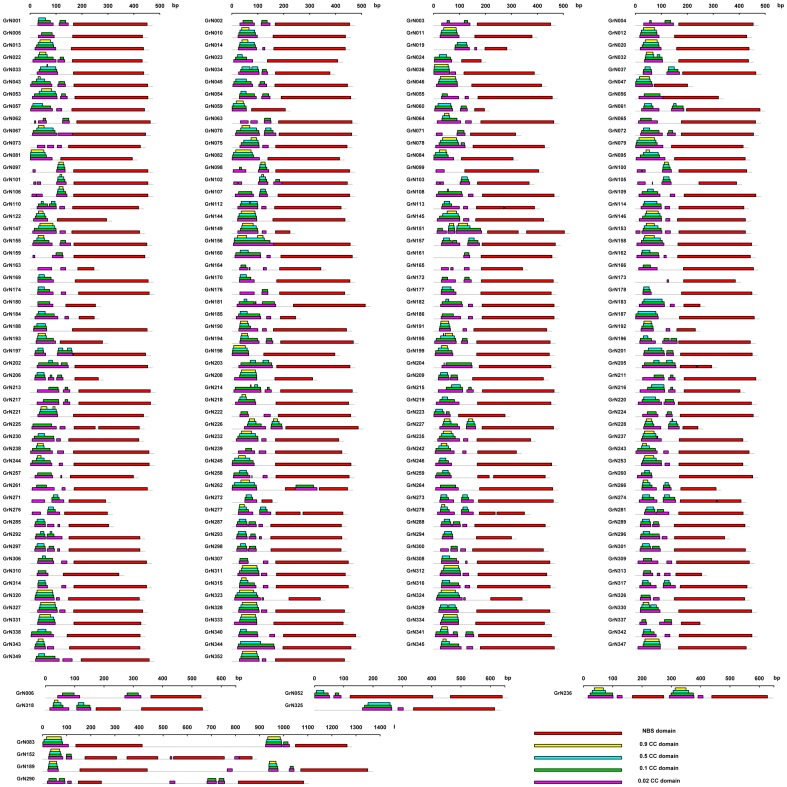
Number of NBS-encoding resistance genes with CC domain in *G. raimondii* under different thresholds of MARCOIL. Gray lines represent NBS-encoding resistance genes, and the different color boxes above the genes indicate the locations of CC domains under different thresholds.

For both regular and non-regular NBS genes, we found that when the threshold value increases, the number of genes containing CC domain decreases. Four thresholds were set to detect CC domains: 0.02, 0.10, 0.50, and 0.90; the numbers of genes detected containing CC domains for each threshold value were: 219, 214, 176, and 91, respectively. The lengths of CC domains detected under a higher threshold were always shorter than the lengths of CC domains detected under a lower threshold. Moreover, the regions detected under a higher threshold were always included by the regions detected under a lower threshold (Figure 1). This indicates that there is a high diversity among the N-terminal CC domains for cotton NBS resistance genes.

Under the lowest threshold (0.02), 219 of the regular and non-regular NBS genes were detected containing CC domains; only 91 (41.36%) of these genes could be detected having CC domains under a threshold of 0.9, which means that the CC domains of the other 128 genes have more N-termini structural diversity. When using MEME to detect the motifs for the CC domains of the 128 genes, we found that: no motif was detected as being shared by all of the genes; in fact, no detected motif was shared by more than 50 genes (Figure S1, A). When the CC domains for the 91 genes were detected, we also found that no motif was shared by all of the 91 genes; in fact, no motif was shared by more than 50 genes (Figure S1, B). In conclusion, this motif analysis of the CC domains indicates that no motif was conserved by all of the CC domains no matter what threshold was used. Again, this implies that there is a high diversity among the N-terminal CC domains in cotton NBS resistance genes.

### Genomic locations and duplication of NBS genes

**Figure 2:**
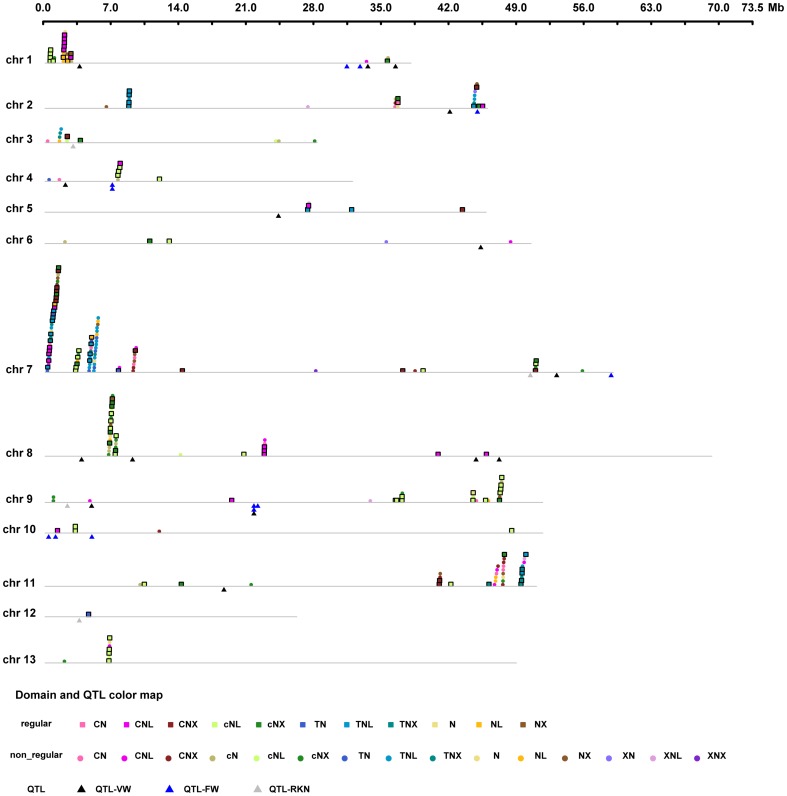
Distribution of NBS-encoding resistance genes in *G. raimondii*. Gray lines represent all 13 chromosomes in the diploid cotton. Boxes indicate the locations of the regular NBS-encoding genes, while stars indicate the locations of the non-regular NBS-encoding genes (Boxes and stars are always above the chromosomes). The triangles below the chromosomes indicate the locations of the disease resistance QTL that is within the 3-Mb flanking region of the NBS genes. QTL-VW means *Verticillium* wilt QTL; QTL-FW means *Fusarium* wilt; QTL-RKN means root-knot nematode resistance QTL.

Based on the location of individual NBS genes, 265 of the 355 NBS-encoding genes were mapped on the 13 chromosomes, while the remaining genes were located on another 34 sequences that have not been yet linked to a chromosome (Table S1). The number of NBS-encoding genes distributed on chromosomes 1 to 13 were 24, 22, 11, 8, 5, 5, 87, 32, 23, 5, 34, 1, and 8 (Table 2); another 15, 14, 10, and 7 NBS-encoding genes were distributed on scaffold377, scaffold461, scaffold406, and scaffold439, respectively; the left 44 NBS-encoding genes were distributed on the other 30 scaffolds, with no more than 3 genes per scaffold. Figure 2 shows the chromosomal locations of the NBS genes. Apparently, the distribution of NBS-encoding genes and gene types was nonrandom in the diploid cotton (Figure 2 and Table S1). For example, chromosome 7 contained not only the greatest number of NBS-encoding genes, but also the greatest variation in gene types (e.g. CNL, TNL, XNL, NL, TN, and so on, were all involved in chromosome 7), whereas only one NBS-encoding gene was located on chromosome 12.

**Table 2:** NBS-encoding resistance genes genome location in *G. raimondii*.

Chromosome or scaffold	Number of NBS genes	Number of genes in clusters	Number of genes with duplication events	Numbers of duplicated genes in clusters
**chromosome 1**	24	23	0	0
**chromosome 2**	22	18	2	2
**chromosome 3**	11	6	1	0
**chromosome 4**	8	5	0	0
**chromosome 5**	5	3	1	0
**chromosome 6**	5	0	0	0
**chromosome 7**	87	81	22	21
**chromosome 8**	32	28	12	12
**chromosome 9**	23	17	1	0
**chromosome 10**	5	2	0	0
**chromosome 11**	34	28	6	6
**chromosome 12**	1	0	0	0
**chromosome 13**	8	7	5	5
**scaffold377**	15	15	7	7
**scaffold461**	14	14	7	7
**scaffold406**	10	10	4	4
**scaffold439**	7	7	2	2
**other scaffolds**	44	24	5	4
**Total**	355	288	75	70 (93.3%)

Studies of NBS-encoding genes in Arabidopsis, rice, sorghum, and maize have shown uneven chromosomal distributions on them; moreover, the studies also shown that most NBS-encoding genes are found in clusters [3], [10], [16], [17]. In this research, a gene cluster was considered a chromosome or a scaffold region when such region contained two or more genes within 200 kb [47]. Of the genes analyzed in this research, 288 were located in 49 gene clusters (Table S1), each with an average of 6 genes. Among the 49 clusters, seven of these were located on chromosome 7, six were located on chromosome 1, five were located on chromosomes 9, four clusters per chromosome were located on chromosomes 2, and 11, three were located on chromosome 8, two were located on chromosome 3, and only one cluster was found on chromosomes 4, 5, 10, and 13. No clusters were found on chromosome 6 and 12. The remaining 14 clusters were located on scaffolds. Most gene clusters contained more than three genes; the largest cluster contained 32 NBS genes on chromosome 7. 85.97% of the diploid cotton NBS genes fell into gene clusters (Figure 2 and Table S1), similar behavior with that of Arabidopsis, rice, sorghum, and maize. It is important to mention that this percentage was determined by neglecting 20 NBS genes; of these genes, one was located on chromosome 12 and the remaining 19 were located on 19 scaffolds (*i.e.* one gene per chromosome or one gene per scaffold).

According to the same parameters of gene duplication used by Gu *et al.* [37], 74 NBS-encoding genes with duplication events were detected and subsequently divided into 23 gene families including 16 gene pair families and 7 gene families that contain at least 3 members (Table S2). When the frequency of duplication events dependence on the numbers of genes per location (a chromosome or a scaffold) was investigated, we found that locations containing more NBS-encoding genes have higher number of duplication events (Table 2), which indicates that tandem duplication is a main factor contributing to the expansion of the NBS genes. For example, among the 13 chromosomes, chromosome 7 was detected containing the most NBS-encoding genes (87), and 22 of them were detected in the duplication events. Furthermore, among the duplication events, 93.24% (69/74) (Table 2) of the NBS were located in clusters. In comparison with these obvious tandem duplication events, only one gene family (family8_3) was involved in chromosome-level duplication events (Table S2), which also suggests that tandem duplication rather than chromosome-level duplication plays a major role in the duplication of NBS genes. It should be noted that families containing members located on scaffolds were not accounted in chromosome-level duplication events, because the locations of these family members still not aligned into any chromosomes.

Just a few number of regular NBS-encoding genes (CNL, TNL, or both CNL and TNL) could be detected in most of the chromosomes. In comparison with the regular CNL or TNL gene subgroups, the rest of the regular or non-regular NBS gene subgroups could be detected in major quantities per chromosome. Furthermore, the regular NBS-encoding genes were often surrounded by non-regular NBS-encoding genes, and longer NBS-encoding genes were often surrounded by shorter NBS-encoding genes (Figure 2 and Table S1). In order to investigate the phylogenetic relationship between these regular and non-regular genes, we constructed two phylogenetic trees. One of the trees was constructed by using the genes belonging to the biggest cluster (cluster 15) of chromosome 7 (see Figure S4); these genes included 5 regular TNL genes and 8 non-regular genes with TIR domain. The other tree was constructed by NBS genes from chromosome 8 (Figure S5) that included 5 regular CNL genes and the genes of the biggest cluster (cluster 22). The phylogenetic tree of chromosome 7 showed that all non-regular TNL, TNX, or TN genes (black genes in Figure S4) of cluster 15 likely formed a small clade with any of those regular TNL genes (blue genes in Figure S4), and the non-regular genes of such a clade were usually shorter than the regular genes having the same most recent common ancestor as the clade. The phylogenetic tree of chromosome 8 showed a similar behavior as that one for chromosome 7, all the non-regular cNL, cNX, or cN genes (black genes in Figure S5) in cluster 22 likely formed a small clade with any of those regular cNL or cNX genes (green genes in Figure S5), and the non-regular genes contained in such a clade were usually shorter than the regular genes with the same most recent common ancestor as the clade; furthermore, the phylogenetic tree of chromosome 8 also showed that all the cNL or cNX genes have the same ancestor as regular CNL gene GrN195. According to the above two phylogenetic tree examples, we inferred that those non-regular or shorter NBS genes should evolve from regular CNL or TNL genes.

### Phylogenetic analysis of NBS-encoding genes

**Figure 3:**
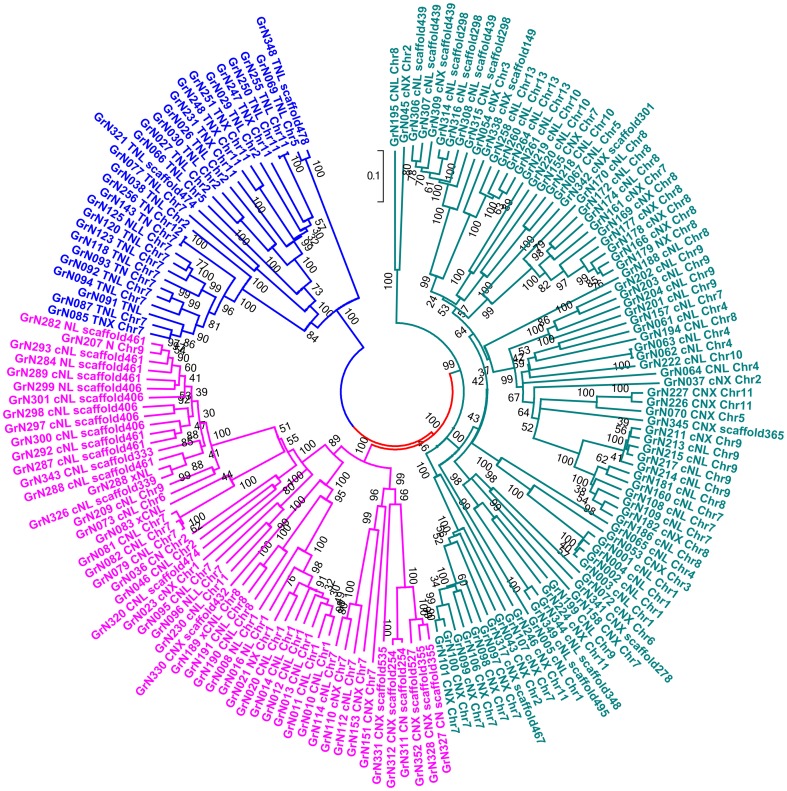
Phylogenetic tree derived from NBS-encoding resistance genes in *G. raimondii*. The neighbor-joining tree was constructed using the sequences of 163 regular NBS containing proteins in *G. raimondii*. Bootstrap values are indicated on the branches. Each *G. raimondii* protein is encoded by its name, and then followed by its type (CNL, TNL, TN and so on) and location (chromosomes or scaffolds). Blue clades are genes with TIR domain, while pink and green clades are genes with CC domain.

The amino acid sequences of the NBS domain of the 163 regular diploid cotton NBS-encoding genes were aligned, and a phylogenetic tree was generated by the Neighbor-joining method (Figure 3).

Phylogenetic reconstruction of diploid cotton NBS-encoding genes showed that genes located in the same chromosome or the same scaffold were most likely found in the same clade. According to this, we further confirmed that tandem duplication rather than segmental duplication plays a major role in the duplication of NBS-encoding genes.

As in Arabidopsis, the phylogenetic tree of the diploid cotton NBS domain also contained two major clades, which suggests that the evolutionary pattern of the diploid cotton NBS domain was similar to that of Arabidopsis. One major clade of the phylogenetic tree was formed by the NBS genes with TIR-motif, and the other clade was mainly formed by the NBS genes with CC domains; this result is consistent with previous studies in different species [11]–[14], [17]. In the case of the CC domain NBS clade, another two clades were differentiated from this clade, which suggests a complex pattern of evolution of CC domain NBS genes. The phylogenetic tree also showed that the NBS genes detected with CC domains under thresholds lower than 0.9 were also included in the CC domain clades, which suggests that these genes share the same origin as those NBS genes detected with CC domains under a threshold of 0.9.

For comparative purposes, we constructed another phylogenetic tree (Figure S6) containing the 163 regular diploid cotton NBS-encoding genes and 71 manually curated resistance NBS-encoding genes from other plants (Table S3). In this phylogenetic tree, all the genes with TIR domains, even if they belong to different species, formed an independent clade excluding any non-TIR genes. The remaining genes, which mainly contained CC domains, formed another two clades. Although most of the genes from the same species were found clustering together, several dicotyledon (purple bracket in Figure S6) and one monocotyledon (green bracket in Figure S6) resistant NBS-encoding genes shared similarity, and formed clusters with the diploid cotton NBS-encoding genes. In the TIR clade, two genes formed a small clade with two *Linum usitatissimum* Flax rust disease resistance genes. In the case of CC I clade, three genes belonging to chromosome 5, 7, and 10, respectively, formed a small clade with the Potato Late Blight disease resistance gene *Rpi-blb1*; four genes from chromosome 9 were clustered with two Soybean *Phytophthora* root disease resistance genes (*Rps1-k-1* and *Rps1-k-2*); six genes, four from chromosome 1, one from chromosome 3, and one from chromosome 4, showed similarity to the *Cucumis melo Fusarium* wilt disease resistance gene *FOM-2*. In CC II clade, four genes belonging to chromosome 7 clustered in a small clade with the *Cucumis melo* Melon aphid disease resistance gene *VAT*; another thirteen genes belonging to chromosome 1, 8 and scaffold432 formed a clade with two *Arabidopsis thaliana* Bacterial Blight disease resistance genes (*RPS5* and *Rps2*).

### Disease resistance QTL adjacent to NBS-encoding genes

In cotton, some disease resistance associated quantitative trait loci (QTL) have been identified [40]–[45]. These QTL were associated with the resistance to *Verticillium* wilt, *Fusarium* wilt, and Root-Knot Nematode. Totally, 98 markers (Table S4) were detected corresponding to these QTL. To detect the co-localization of those QTL and the NBS genes, we analyzed the locations of these QTL in the diploid cotton genome based on the information of their corresponding markers.

Among the 98 markers, 74 markers can be located on the diploid cotton. Among the 74 markers, 32 were located within 3-Mb flanking region of the genes, 23 were located further away from 3-Mb region of the NBS genes, 17 were located on scaffolds containing no NBS genes, but these scaffolds are no larger than 2MB, therefore, there is a possibility that any NBS genes could be present near any of these 17 markers if the scaffolds were aligned into chromosomes. The last 2 markers were located on scaffolds, which also are no larger than 2MB, but they contain NBS genes.

Most of the markers located within the 3-Mb flanking region of NBS genes were adjacent to the NBS clustered regions (Table 3, Figure 2). For example, the clustered regions of: NBS genes on chromosome 8 harbored 3 QTL conferring *Verticillium* wilt resistance. In the case of chromosome 1, 2, 3, 4, 5, and 7, *Verticillium* wilt, *Fusarium* wilt and Root-Knot Nematode resistance QTLs were found within the 3-Mb flanking region of NBS gene clusters. QTLs located outside of the 3-Mb flanking region of an NBS may be linked with other non-NBS resistance genes.

**Table 3:** Correlation between NBS-encoding resistance genes and disease resistance QTL, as well as their locations in *G. raimondii*.

Chromosome or Scaffold	Number of NBS genes	Number of QTL	Number of QTL within 3-Mb flanking region of NBS genes	Number of QTL out 3-Mb flanking region of NBS genes
**chromosome 1**	24	7	5	2
**chromosome 2**	22	5	2	3
**chromosome 3**	11	2	1	1
**chromosome 4**	8	3	3	0
**chromosome 5**	5	2	1	1
**chromosome 6**	5	6	1	5
**chromosome 7**	87	4	3	1
**chromosome 8**	32	8	5	3
**chromosome 9**	23	8	6	2
**chromosome 10**	5	6	3	3
**chromosome 11**	34	2	1	1
**chromosome 12**	1	2	1	1
**chromosome 13**	8	0	0	0
**scaffolds**	90	19	2	
**total**	355	74	34	23

Since those markers were derived from tetraploid cotton species, we compared the locations of the 74 markers between the diploid cotton genome and the tetraploid cotton genome. In tetraploid cotton, chromosome numbers 1 through 13 are reserved for the A subgenome and chromosome numbers 14 through 26 are reserved for the D subgenome [48].Based on this Rule, chromosome 14 through chromosome 26 were defined as Dt subgenome in tetraploid cottons, because they derived from D genome. According to this definition, 61 markers have locations on Dt subgenome in tetraploid cotton (Table S4), which suggest that the corresponding QTL should also have infections on the diploid cotton too; the other 13 markers have locations only on At subgenome in tetraploid cotton, we inferred this may be the result of sequence conversion from Dt to At subgenome.

For the 24 markers that could not be located on the diploid cotton genome, we also checked for their possible location in the genome of tetraploid cotton. We found that 11 of them have locations only on At subgenome and we also inferred that the corresponding QTL of these markers may be originated from A diploid cotton. The other 13 markers have locations on Dt subgenome or both, and we inferred that: these kinds of markers may not be general in the diploid cotton, or their relative sequence was converted from subgenome At to Dt in tetraploid cotton.

## Discussion

Cotton (*Gossypium spp*.) is one of the most economically important crops that provides the world's leading natural fiber. Diseases, such as *Fusarium* wilt and *Verticillium* wilt are the main threats to crop production, so disease resistant breeding is one of the most important purposes of cotton breeding programs. Increasing the knowledge of the distribution, structure and organization of disease resistant genes will help the breeders to improve crop's disease resistance. The availability of the cotton genome sequence provides us the opportunity to identify the NBS-encoding resistance gene homologs, and also provide a potential opportunity to select cotton disease resistant-related NBS-encoding genes. In this study, by analyzing the motifs, the structural diversity, the chromosomal distribution, the phylogenetic relationships, and the association between disease resistance QTL and NBS-encoding genes, we not only confirmed some features of NBS-encoding genes that have been already observed in other plant species, but also elucidated some features that are different from those of other plants.

### N-terminal diversity of NBS resistance gene in cotton

In comparison to other plants that already have genome wide NBS-encoding resistance gene identification and analysis [3], [4], [7], [9]–[14], [16], [17], [24], [26], [49], the most distinct feature in *G. raimondii* is the high diversity domains at the N-termini of these NBS resistance genes.

In most of dicot plants, the genes that contain CC domain at their N-termini account for one of the largest proportion of the NBS-encoding resistance genes. For *G. raimondii*, 219 genes were detected containing CC domains when we used the lowest threshold (0.02), but only 91 of them were still detected containing CC domain under a threshold of 0.90. Furthermore, the CC domain sequence lengths detected under a higher threshold were always shorter than those ones detected under a lower threshold, and the CC domain regions detected under a higher threshold were always included by those ones detected under a lower threshold (Figure 1). Furthermore, the motifs analysis of the CC domains indicates that no motif was conserved by all of the CC domains no matter what threshold was used. This indicated that there is a high diversity among the N-terminal CC domains in cotton NBS-encoding resistance genes.

Seventy one of the NBS resistance genes in *G. raimondii* were detected as not containing TIR nor CC domains at their N-termini. Among the 71 genes, 63 genes were classified on N, NL, and NX subgroups, because their sequence before NBS domain contained no more than 100 amino-acid residues. In the case of the 63 genes, none of their N-termini were annotated to any current known domains, and we inferred this kind of genes might not contain a N-terminal domain, because the sequence before NBS domain is too short. The left 8 genes were classified on XN, XNL, and XNX subgroups, because their sequence before NBS domain contained more than 100 amino-acid residues. In the case of the 8 genes, 5 of their N-termini were annotated to current known domains, which suggest that this kind of genes acquired new N-terminal domains that differ with TIR and CC domains. Four of these five N-termini were annotated as Arabidopsis broad-spectrum mildew resistance protein RPW8. Wang et al. [49] reported that RPW8 proteins are atypical resistance proteins that contain a N-terminal transmembrane domain and one to two coiled-coil domains. Moreover, Eckardt [50] confirmed that RPW8 resistance protein is recruited to the extrahaustorial membrane of biotrophic powdery mildew fungi. The last one of the five N-termini was annotated as RNA 3′-terminal phosphate cyclase. Although RNA 3′-terminal phosphate cyclase is a member of a family of RNA-modifying enzymes that are conserved in eukaryotes, bacteria and archaea, the physiological function of this domain is still unclear (http://www.ebi.ac.uk/interpro).

With exception of the TIR domain, the structural diversity that exists among the N-termini of cotton NBS-encoding resistance proteins is remarkable. According to the five annotations of the non-TIR and non-CC N-termini, we inferred that the non-TIR, non-CC, and non-conserved CC N-terminal domains should have similar functions as TIR and CC domains, even if they have a high diversity. Diversifying selection, phenomenon that has been shown in other plants, may cause this high diversity. Diversifying selection gives the plant more specific resistance, or lets the plant gained novel disease resistance. The reason that diversifying selection tends to act on N-termini regions is because the N-termini domains usually have the function of determining the specificity of resistance and signaling.

### NBS domain diversity of NBS resistance gene in cotton

Another distinct feature in the diploid cotton is the high proportion of non-regular NBS genes. Among the 355 resistance genes containing NBS domain, 193 (54.37%) of them were classified as non-regular genes because of the lack of one or more motifs of NBS domain. These non-regular cotton NBS resistance genes have partial motifs of NBS domain likewise pseudogenes found in previous studies on Arabidopsis [51], *Medicago truncatula* [12], and potato [14]. Therefore, we inferred that these non-regular NBS genes may have similar functions with the above pseudogenes, such as the regulation of the messenger-RNA stability on the corresponding homologous genes [52].

One possible explanation for the high number of non-regular NBS-encoding genes is the genome wide deletion of redundant NBS-encoding gene components after whole genome duplication. Similar phenomenon of redundant NBS-encoding gene loss has also been reported in other plant genomes. Gene duplication, including whole genome duplication (WGD), has resulted in a substantial increase in NBS gene number. However, no NBS genes in the WGD blocks of rice [53] and grape [54] were detected, and only a small number of these genes have been identified in Arabidopsis [27] and poplar [54], indicating that most duplicated NBS-encoding genes were lost soon after WGD. The whole genome duplication event was clearly observed in *G. raimondii* genome [31], however, no NBS genes were found in duplication blocks, suggesting that NBS-encoding genes loss also occurred in *G. raimondii* genome.

It is also interesting to note that most of the non-regular NBS genes in *G. raimondii* seem to be formed by truncating motifs from one end or both ends of NBS domain; for example, non-regular NBS genes were found with: Kinase2, Kinase3, GLPL and MHDL motifs (left end P-loop motif truncated), P-loop, Kinase2 and Kinase3 motifs (right end GLPL and MHDL motifs truncated), and Kinase2 and Kinase3 motifs (both left end P-loop, and right end GLPL and MHDL motifs truncated). Cases where the non-regular NBS genes lost the P-loop motif and sequence before NBS domain, or the MHDL motif and sequence after NBS domain were identified too. The above phenomenon indicates that NBS gene loss should begin around any or both ends of NBS domain; this NBS genes loss process promotes a rich number and type of non-regular NBS genes in *G. raimondii.*

### Duplication of NBS resistance genes in cotton

In *G. raimondii*, 85.97% of the NBS genes fell into gene clusters, this percentage of clustered genes is slightly higher than those ones found on rice [16] and Arabidopsis [17] (75.9 and 73.2%, respectively), suggesting that gene duplication, especially tandem duplication, played a more important role for the evolution of the diploid cotton NBS resistance genes than for rice and Arabidopsis NBS resistance genes.

The distribution of NBS genes on chromosomes was considerably variable. In addition, duplication of NBS-encoding genes was found to promote higher number of genes in the chromosomes (table 2). For example, 20 genes out of the 87 NBS genes belonging to chromosome 7 were involved in duplication events, while only 1 gene out of the 5 NBS genes belonging to chromosome 5 was involved in duplication events. Among the duplication event genes, 93.3% (70/75) (table 2) of the NBS paralogs were located in clusters. This finding indicates that chromosomal hot spots in which the NBS genes share greater homology [10] also existed in *G. raimondii* genome, and genes in such hot spots may have been expanded by tandem duplication.

Gene duplication is an important mechanism for the evolution of divergent protein functions and provides sources of species-specific resistance genes with taxonomically restricted functionality. As mentioned in the above paragraphs, in *G. raimondii*, there is high diversity on the N-termini domains and NBS domains of these cotton resistance genes, we inferred that gene duplication, especially tandem duplication, is also one possible reason for the production of these diversity (i.e. some of the high diversity of the N-termini domains may be produced during gene duplication procedures and some of the high diversity of NBS domains may be caused by partial sequence loss of NBS-encoding genes during gene duplication).

### Phylogenies of NBS resistance genes in cotton

By previous studies, NBS-encoding genes with TIR domain have been detected having two unique characteristics: 1) this kind of genes always forms an independent clade excluding any non-TIR genes [12]–[14], and 2) such clade also forms species-specific TIR clades among different species [54]. These two characteristics suggest that TIR-NBS genes not only are different from non-TIR genes, but also can serve as tools to differentiate among different species.

These characteristics were also detected in the present study. The regular conserved NBS domain of *G. raimondii* and other plants were used to study the Phylogenetic topology architecture of this gene family. Phylogenetic analysis showed that NBS gene with TIR-domain formed an independent clade away from non-TIR genes. Within such TIR clade, species-specific clades were also detected as expected. This result suggests that in *G. raimondii* the TIR genes evolution process is different from those of non-TIR genes and TIR genes from other plants; this difference on the evolution processes should be used to recognize cotton species-specific pathogens.

Another characteristic of these cotton TIR NBS genes is that most of them were concentrated on a small number of clusters, indicating that this kind of genes in *G. raimondii* were increased likely by tandem duplication.

### The correlation between disease resistance QTL and NBS resistance genes in cotton

Genome sequencing, physical alignment of genomic regions into chromosomal maps and the anchoring of genetic maps are all steps that will improve the accuracy of detecting resistance genes underlying quantitative trait loci (QTL) and gene functions of important biological processes in crops [41]. In this study, we detected the genome-wide candidate NBS-encoding resistance genes and analyzed their structure, genome location and duplication, and Phylogenetic topology architecture. We also investigated the correlation between the candidate NBS-encoding genes in G. *raimondii* and the disease resistance QTL that have been detected in tetraploid cotton.

Among the markers corresponding to the disease resistance QTL in tetraploid cotton, 75.5% (74/98) of them can be located on the diploid cotton, suggesting that the tetraploid species and diploid species of cotton share some disease resistance QTL. Moreover, most of cotton diseases, like *Verticillium* wilt and *Fusarium* wilt, are harmful for both tetraploid and diploid species, implying again that these two cotton species likely share disease resistance QTL.

Among the 74 markers that can be located on the diploid cotton, 45.9% (34/74) were located within the 3-Mb flanking region of the NBS-encoding resistance genes. Another 17 markers were located on short (<2 Mb) scaffolds, considering that some of these markers should be located near (in 3-Mb flanking regions) NBS genes if the scaffolds were aligned into the chromosomes, the percentage of disease resistance QTL associated with NBS-encoding resistance genes in *G. raimondii* is likely to exceed 50%. A percentage exceeding 50% agrees with previous studies establishing that more than half of plant resistance genes are NBS-encoding genes [55].

Similar correlation between disease resistance QTL and NBS resistance genes has been found in other plants [56]–[59], indicating that the association between disease resistance QTL and NBS genes might be common in crop species. Therefore, we may be able to integrate multi-disease-resistance genes together by developing molecular markers for NBS genes in order to develop a supper disease resistance cultivated cotton variety.

In conclusion, *G. raimondii* NBS-encoding resistance gene analysis of the motifs, the structural diversity, the chromosomal distribution, the phylogenetic relationships, and the association between disease resistance QTL and NBS genes, as well as the results reported in present study offered valuable insights to understand cotton NBS-encoding gene family. The NBS-encoding genes identified by this study can also be used as a source for the selection of resistance genes for common pathogens of other cotton species, because *G. raimondii* pathogens also infect other cotton species. However, the upcoming sequencing of other cotton species will make possible to conduct a higher level comparison of sequences and genome structure among cotton species to address the next issues that were out of the scope of this research: confirm which is the actual cause of the high diversity of the N-termini and NBS domains, determine the functions of such high diversity domains, and make sure if this high diversity is species-specific for cotton.

## Supporting Information

Figure S1 **CC domain Motifs of NBS-encoding resistance genes in** ***G. raimondii*** **.** A: CC domain motifs that only can be detected under thresholds lower than 0.9; B: CC domain motifs that can be detected under a threshold of 0.9. Fifteen putative motifs were identified by MEME, the block diagram shows the best non-overlapping tiling of motif matches on the sequence, and different motifs are indicated by color. The height of a block gives an indication of the significance of the match, and taller blocks indicate greater significance. The names of all the members from the different subfamilies and combined E-values are shown on the left side. Motif sizes are indicated by the scale bar at the bottom.
(PDF)

Figure S2 **Conserved NBS domain motifs of regular NBS-encoding resistance genes in** ***G. raimondii*** **.** Pink highlighted motifs indicate the conserved NBS domain motifs in regular NBS-encoding genes.
(PDF)

Figure S3 **Conserved NBS domain motifs of non-regular NBS-encoding resistance genes in** ***G. raimondii*** **.** Turquoise highlighted motifs indicate the conserved NBS domain motifs in non-regular NBS-encoding genes.
(PDF)

Figure S4 **Gene phylogenetic tree of the biggest NBS-encoding resistance gene cluster on chromosome 7.** Blue genes indicate the regular NBS-encoding genes with TIR domain, black genes indicate the non-regular NBS-encoding genes with TIR domain, and numbers after gene types indicate the length of these genes.
(TIFF)

Figure S5 **Gene phylogenetic tree of five regular CNL NBS-encoding resistance genes and the biggest cluster on chromosome 8.** Red genes indicate the regular NBS-encoding genes with CC domain under a threshold of 0.9, green genes indicate the regular NBS-encoding genes with CC domain under thresholds lower than 0.9, black genes indicate the non-regular NBS-encoding genes with CC domain under thresholds lower than 0.9, and the number after gene type indicate gene length.
(TIFF)

Figure S6 **Phylogenetic tree of NBS-encoding resistance genes derived from** ***G. raimondii*** **and other plants.** The neighbor-joining tree was constructed using the sequences of 163 regular NBS-encoding genes in *G. raimondii* and another 71 NBS-encoding genes in other plants. Bootstrap values are indicated on the branches. Each *G. raimondii* protein (black) is encoded by its name, and then followed by its type (CNL, TNL, TN and so on) and location (chromosomes or scaffolds); each non cotton plant protein (red) is encoded by its name too, but followed by its species and relative diseases. Blue bracket corresponds to TIR clades; pink brackets correspond to non-TIR clades; purple brackets correspond to *G. raimondii* genes similar to other dicotyledon plant NBS-encoding resistance genes; green bracket correspond to *G. raimondii* genes similar to one of rice NBS-encoding resistance genes.
(PDF)

Table S1 **NBS-encoding resistance gene information of** ***G. raimondii*** **.**
(XLSX)

Table S2 **Gene family information of duplicated NBS-encoding resistance genes in** ***G. raimondii*** **.**
(XLSX)

Table S3 **NBS-encoding resistance gene information of other plants.**
(XLSX)

Table S4 **Disease resistance QTL information of cotton.**
(XLSX)
