# Supplementary figures and images for: Systematic Analysis and Comparison of Nucleotide-Binding Site Disease Resistance Genes in a Diploid Cotton Gossypium raimondii
Source: PLoS One. 2013 Aug 6;8(8):e68435. doi: 10.1371/journal.pone.0068435 (PMC3735570; doi:10.1371/journal.pone.0068435)

A

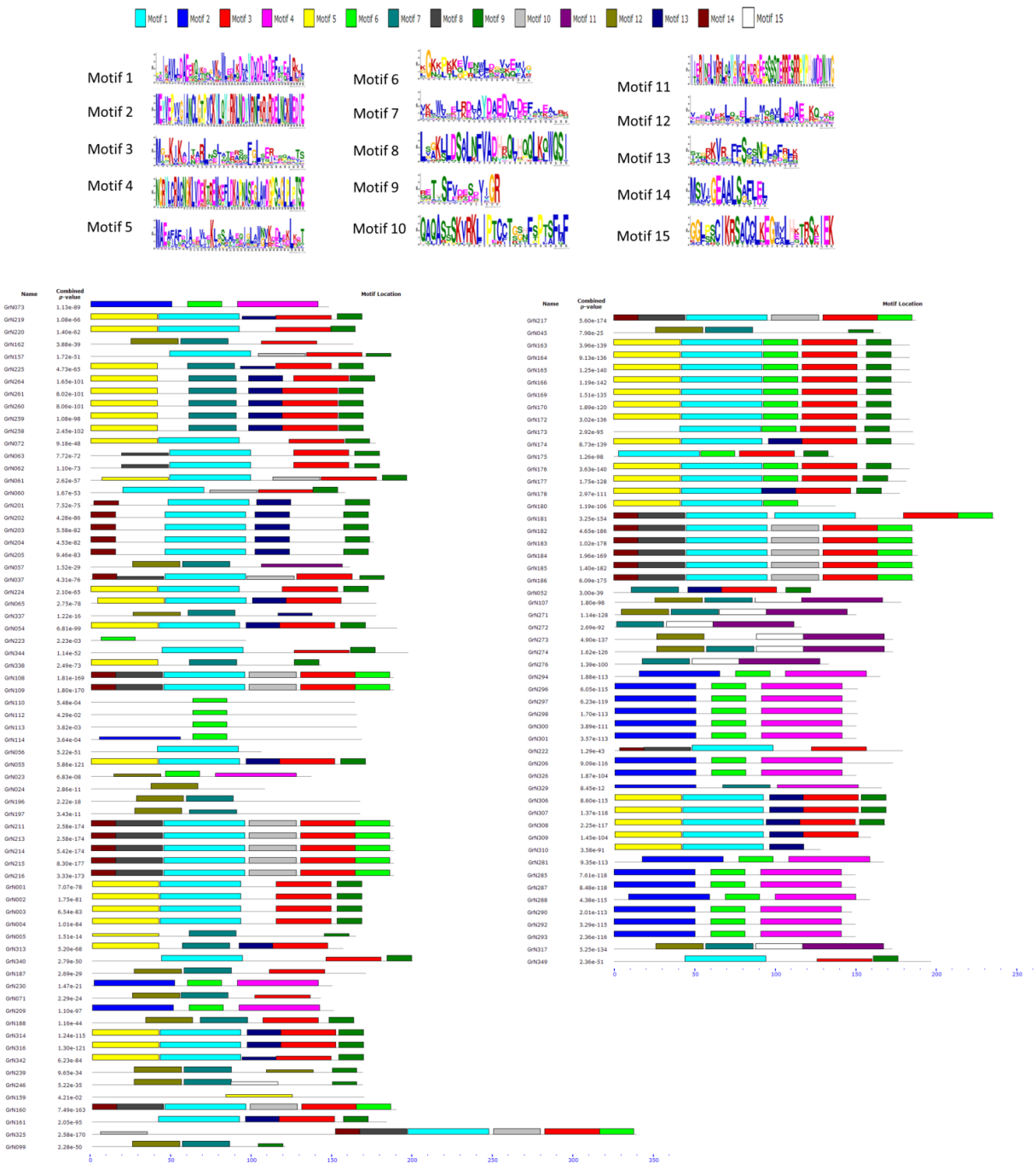

B

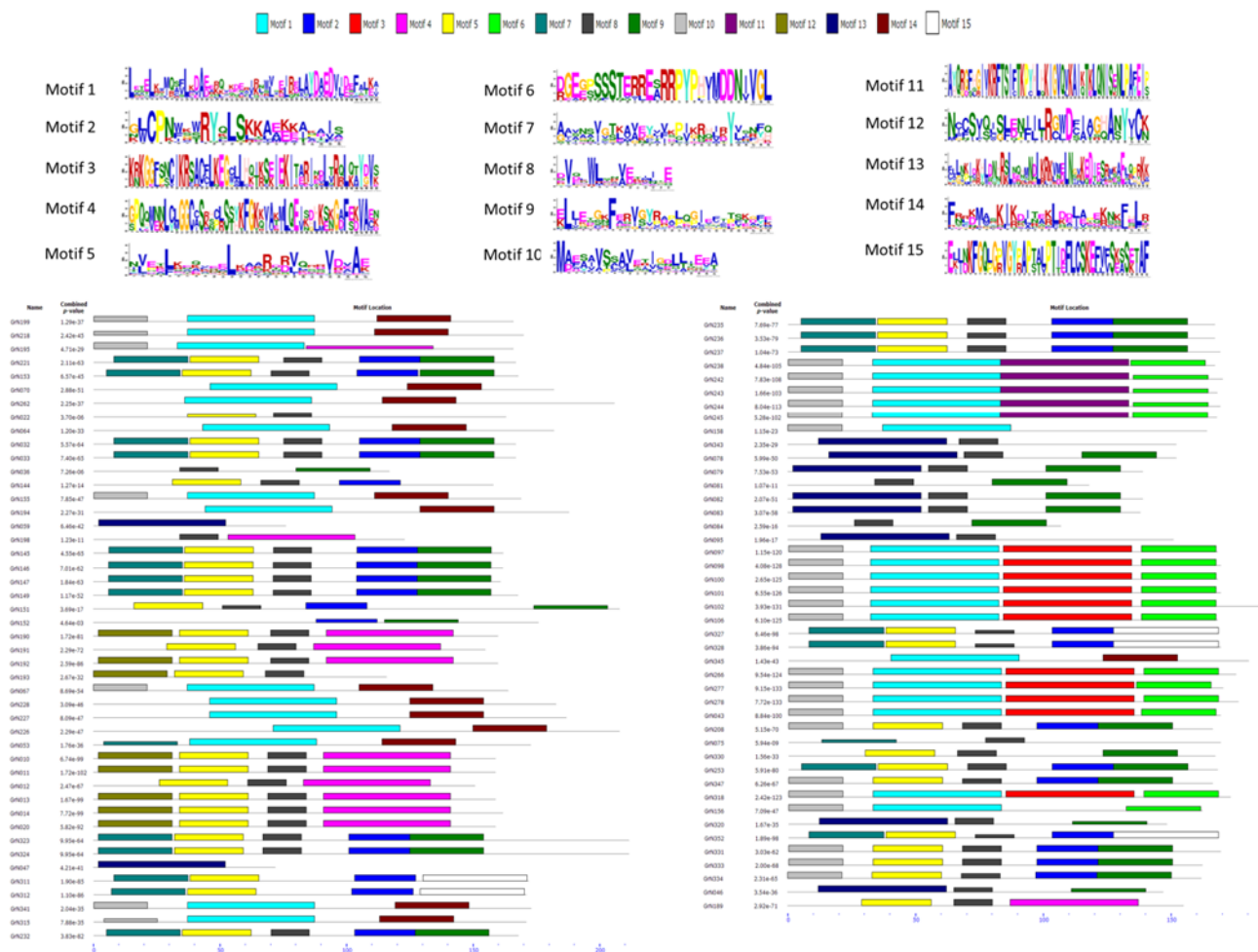

Supplement: Figure S1 — CC domain Motifs of NBS-encoding resistance genes in G. raimondii . A: CC domain motifs that only can be detected under thresholds lower than 0.9; B: CC domain motifs that can be detected under a threshold of 0.9. Fifteen putative motifs were identified by MEME, the block diagram shows the best non-overlapping tiling of motif matches on the sequence, and different motifs are indicated by color. The height of a block gives an indication of the significance of the match, and taller blocks indicate greater significance. The names of all the members from the different subfamilies and combined E-values are shown on the left side. Motif sizes are indicated by the scale bar at the bottom. (PDF) [file pone.0068435.s001.pdf]

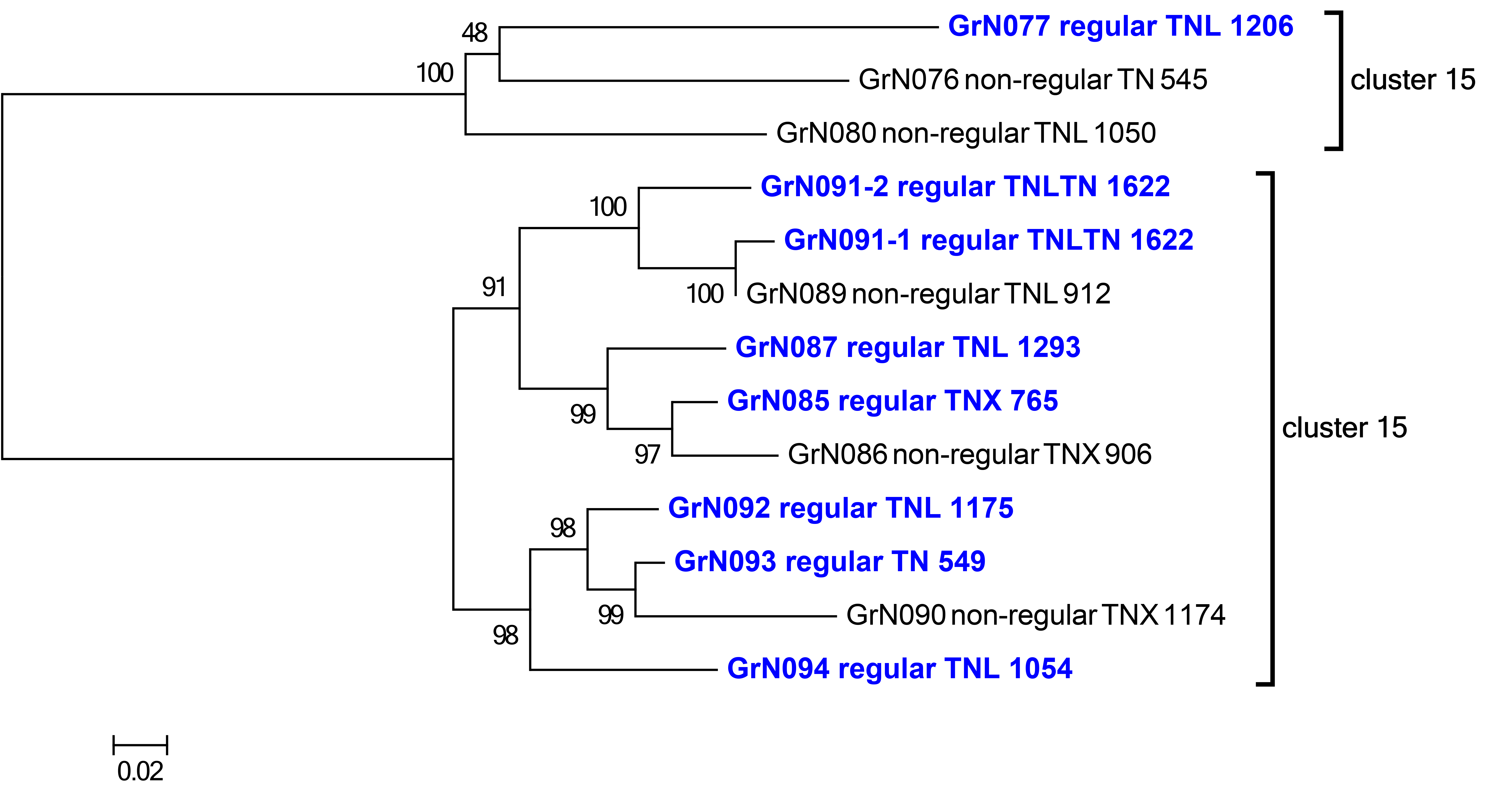

Supplement: Figure S4 — Gene phylogenetic tree of the biggest NBS-encoding resistance gene cluster on chromosome 7. Blue genes indicate the regular NBS-encoding genes with TIR domain, black genes indicate the non-regular NBS-encoding genes with TIR domain, and numbers after gene types indicate the length of these genes. (TIFF) [file pone.0068435.s004.tiff]

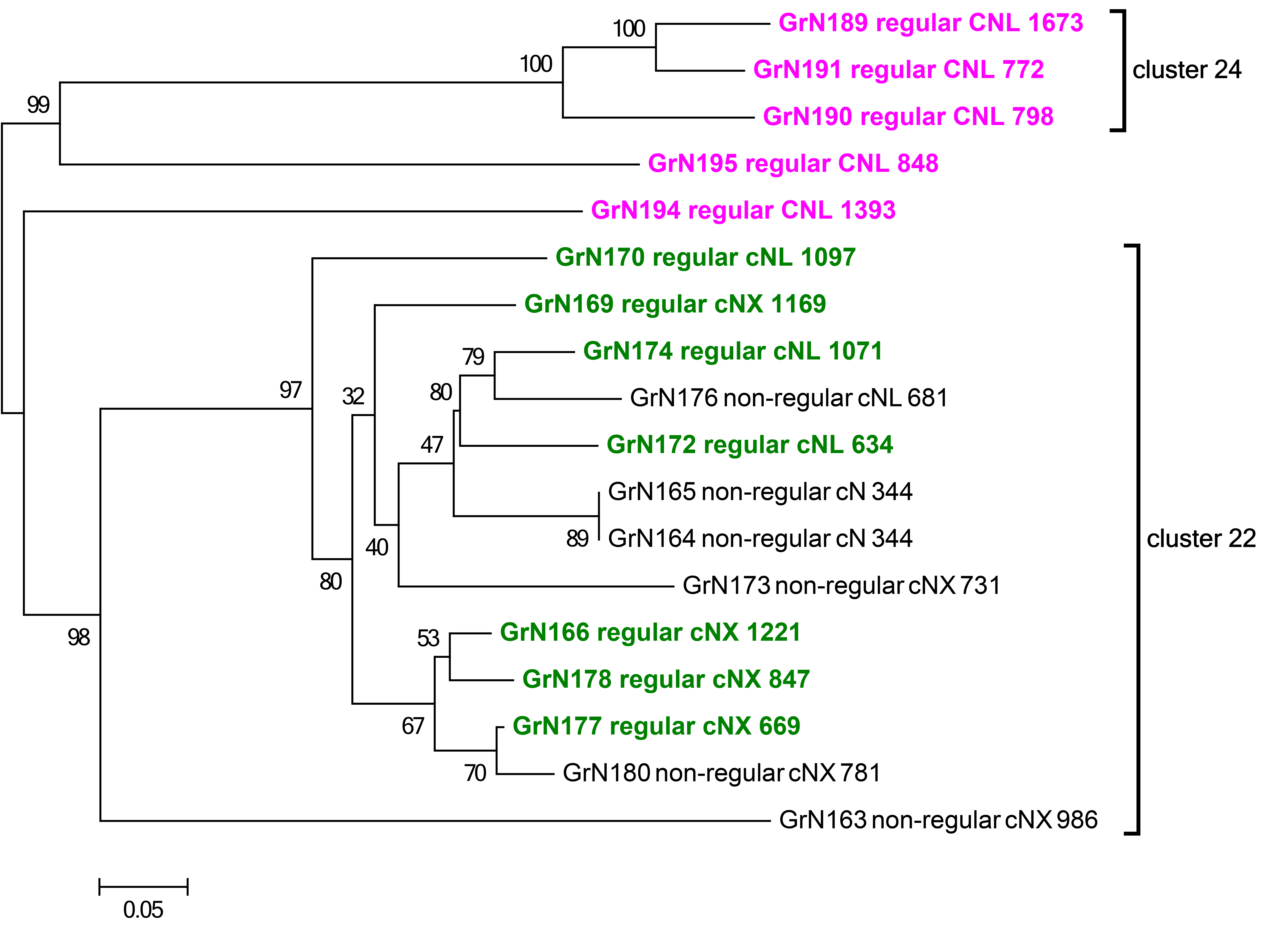

Supplement: Figure S5 — Gene phylogenetic tree of five regular CNL NBS-encoding resistance genes and the biggest cluster on chromosome 8. Red genes indicate the regular NBS-encoding genes with CC domain under a threshold of 0.9, green genes indicate the regular NBS-encoding genes with CC domain under thresholds lower than 0.9, black genes indicate the non-regular NBS-encoding genes with CC domain under thresholds lower than 0.9, and the number after gene type indicate gene length. (TIFF) [file pone.0068435.s005.tiff]

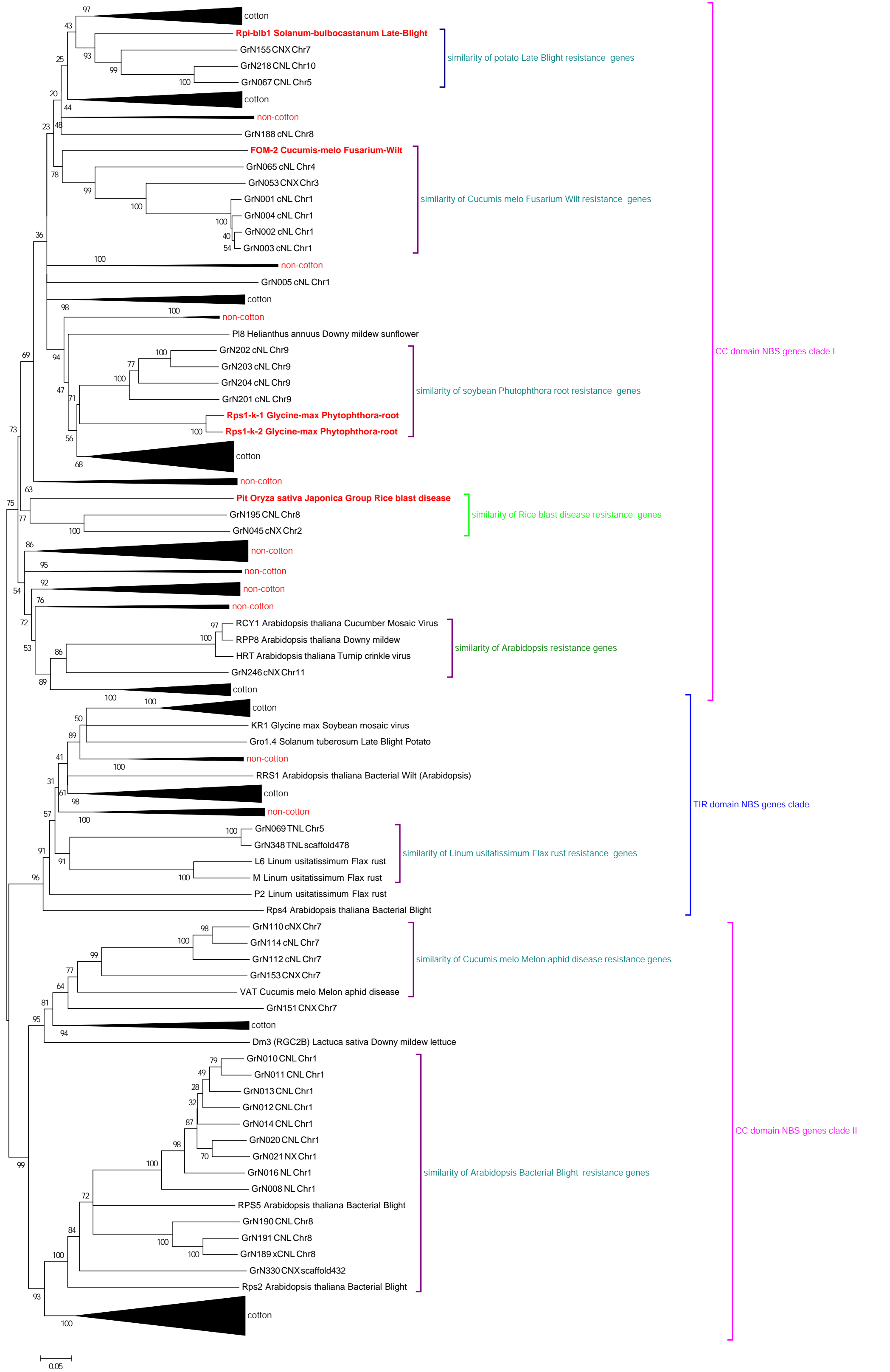

Supplement: Figure S6 — Phylogenetic tree of NBS-encoding resistance genes derived from G. raimondii and other plants. The neighbor-joining tree was constructed using the sequences of 163 regular NBS-encoding genes in G. raimondii and another 71 NBS-encoding genes in other plants. Bootstrap values are indicated on the branches. Each G. raimondii protein (black) is encoded by its name, and then followed by its type (CNL, TNL, TN and so on) and location (chromosomes or scaffolds); each non cotton plant protein (red) is encoded by its name too, but followed by its species and relative diseases. Blue bracket corresponds to TIR clades; pink brackets correspond to non-TIR clades; purple brackets correspond to G. raimondii genes similar to other dicotyledon plant NBS-encoding resistance genes; green bracket correspond to G. raimondii genes similar to one of rice NBS-encoding resistance genes. (PDF) [file pone.0068435.s006.pdf]
