# Supplementary material for: Systematic Analysis and Comparison of Nucleotide-Binding Site Disease Resistance Genes in a Diploid Cotton Gossypium raimondii
Source: PLoS One. 2013 Aug 6;8(8):e68435. doi: 10.1371/journal.pone.0068435 (PMC3735570; doi:10.1371/journal.pone.0068435)
Supplement: Figure S2 — Conserved NBS domain motifs of regular NBS-encoding resistance genes in G. raimondii . Pink highlighted motifs indicate the conserved NBS domain motifs in regular NBS-encoding genes. (PDF) [file pone.0068435.s002.pdf]

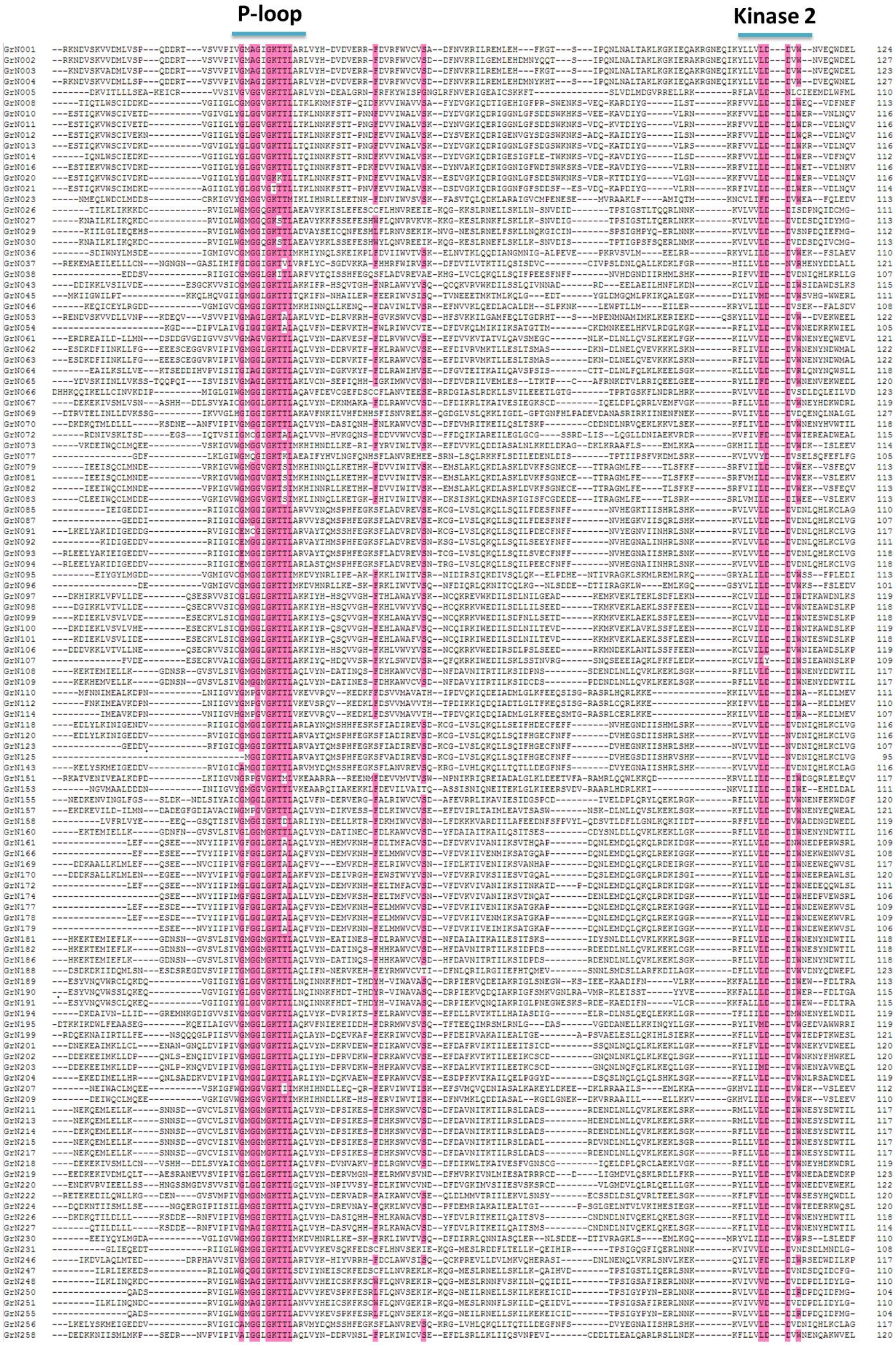
P-loop
Kinase 2

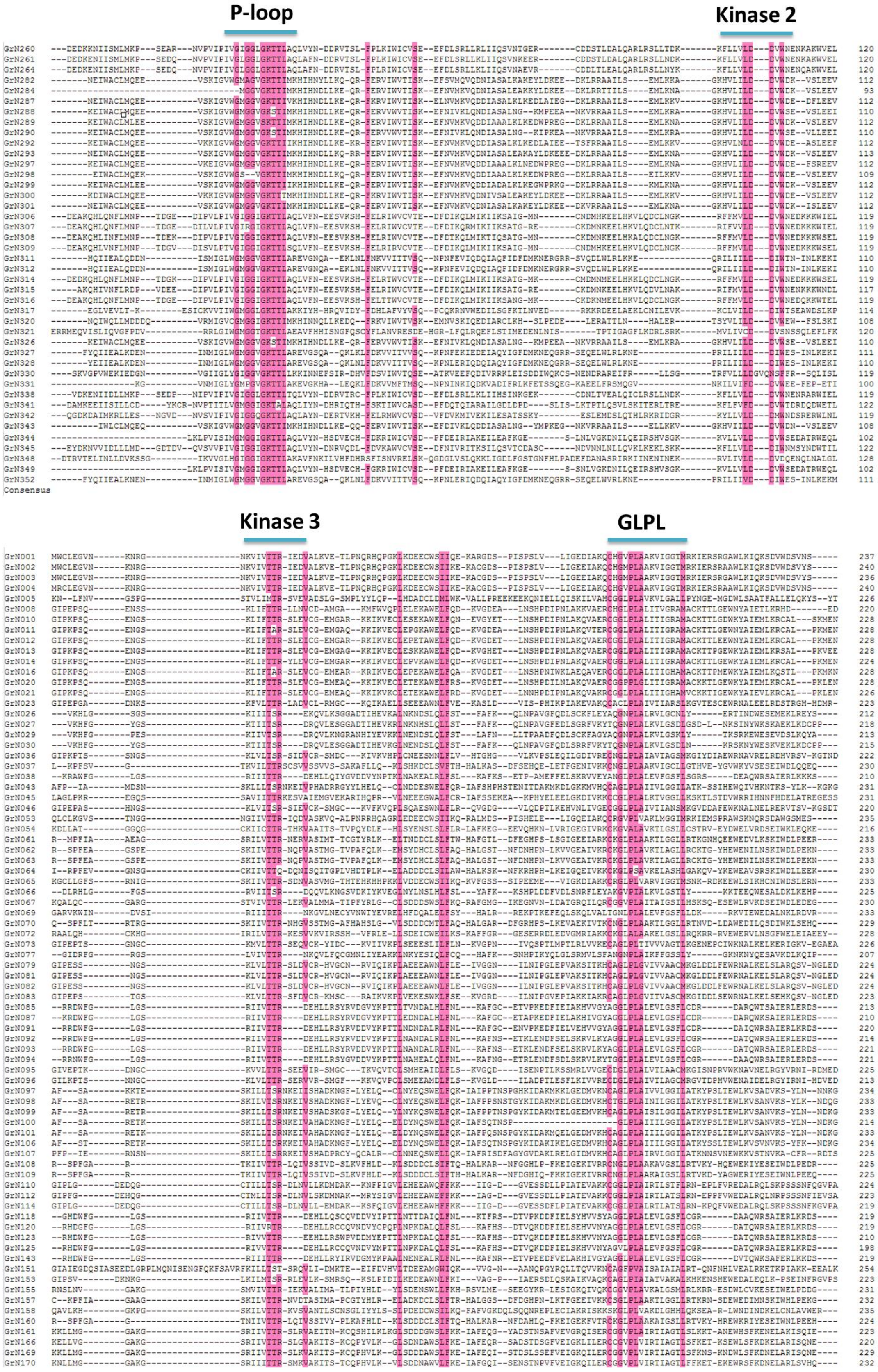
P-loop
Kinase 2
Kinase 3
GLPL

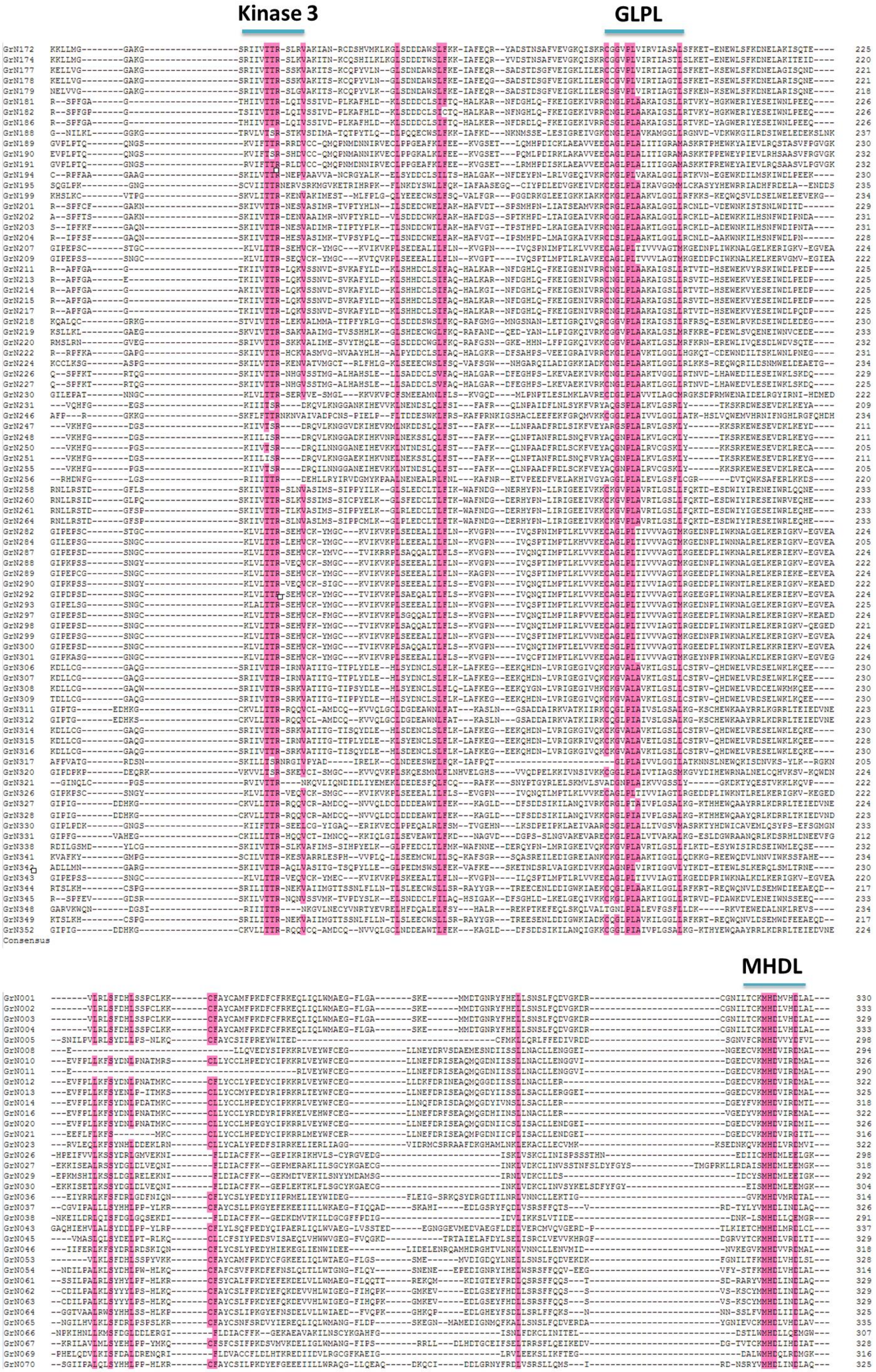
Kinase 3
GLPL
MHDL

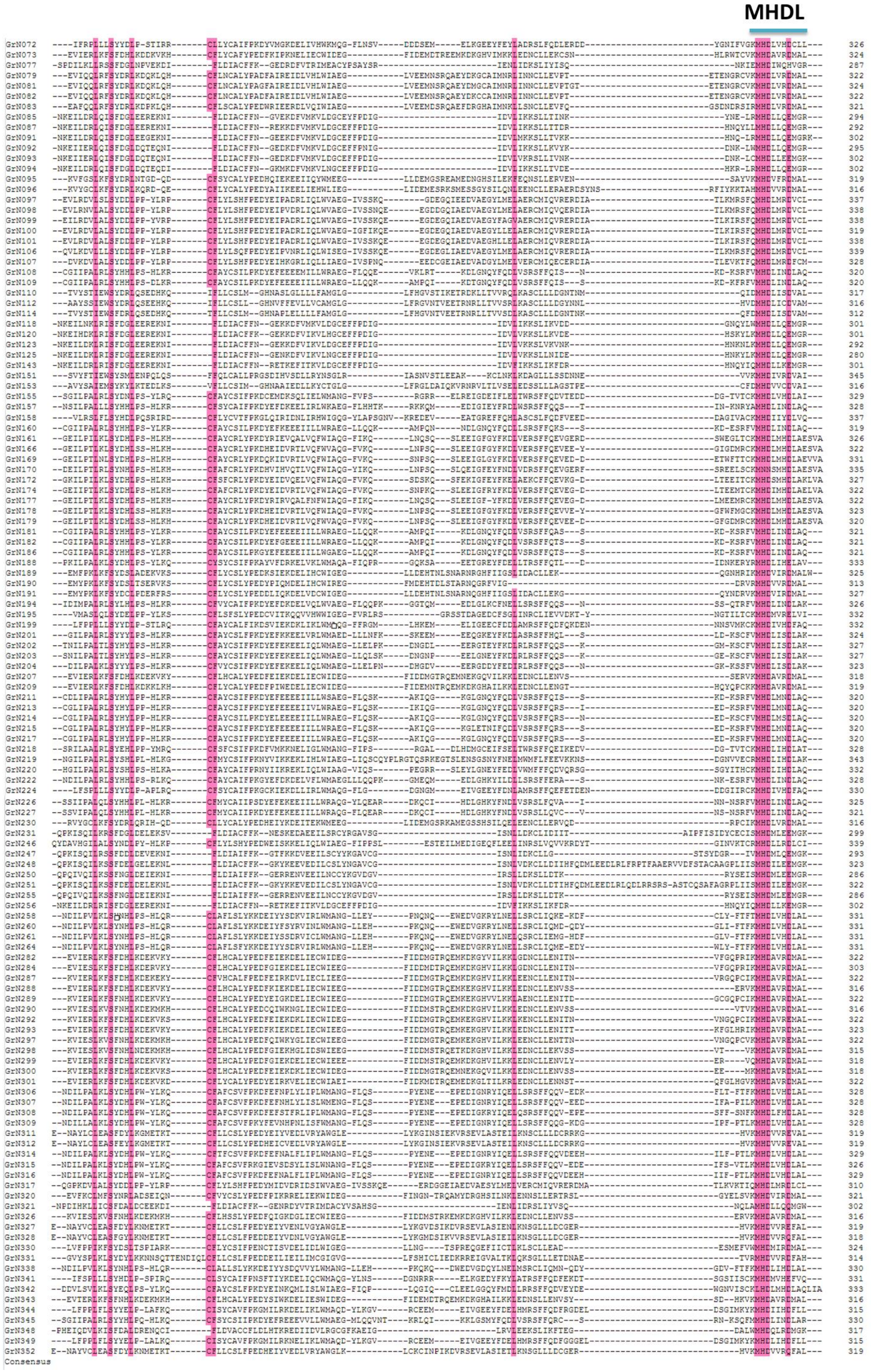
MHDL
